# Towards core outcome set (COS) development: a follow-up descriptive survey of outcomes in Cochrane reviews

**DOI:** 10.1186/s13643-015-0060-0

**Published:** 2015-05-19

**Authors:** Francesca Wuytack, Valerie Smith, Mike Clarke, Paula Williamson, Elizabeth Gargon

**Affiliations:** School of Nursing & Midwifery, Trinity College Dublin, 24 D’Olier Street, Dublin 2, Ireland; School of Nursing & Midwifery, NUI Galway, Galway, Ireland; All-Ireland Hub for Trials Methodology Research, Queens University Belfast, Grosvenor Road, Belfast, BT12 6BA Northern Ireland UK; Department of Biostatistics, University of Liverpool, Crown Street, Liverpool, L69 3BX UK

**Keywords:** Core outcome set, Systematic review, Outcome reporting bias

## Abstract

**Background:**

A core outcome set (COS) can address problems of outcome heterogeneity and outcome reporting bias in trials and systematic reviews, including Cochrane reviews, helping to reduce waste. One of the aims of the international Core Outcome Measures in Effectiveness Trials (COMET) Initiative is to link the development and use of COS with the outcomes specified and reported in Cochrane reviews, including the outcomes listed in the summary of findings (SoF) tables. As part of this work, an earlier exploratory survey of the outcomes of newly published 2007 and 2011 Cochrane reviews was performed. This survey examined the use of COS, the variety of specified outcomes, and outcome reporting in Cochrane reviews by Cochrane Review Group (CRG). To examine changes over time and to explore outcomes that were repeatedly specified over time in Cochrane reviews by CRG, we conducted a follow-up survey of outcomes in 2013 Cochrane reviews.

**Methods:**

A descriptive survey of outcomes in Cochrane reviews that were first published in 2013. Outcomes specified in the methods sections and reported in the results section of the Cochrane reviews were examined by CRG. We also explored the uptake of SoF tables, the number of outcomes included in these, and the quality of the evidence for the outcomes.

**Results:**

Across the 50 CRGs, 375 Cochrane reviews that included at least one study specified a total of 3142 outcomes. Of these outcomes, 32 % (1008) were not reported in the results section of these reviews. For 23 % (233) of these non-reported outcomes, we did not find any reason in the text of the review for this non-report. Fifty-seven percent (216/375) of reviews included a SoF table.

**Conclusions:**

The proportion of specified outcomes that were reported in Cochrane reviews had increased in 2013 (68 %) compared to 2007 (61 %) and 2011 (65 %). Importantly, 2013 Cochrane reviews that did not report specified outcomes were twice as likely to provide an explanation for why the outcome was not reported. There has been an increased uptake of SoF tables in Cochrane reviews. Outcomes that were repeatedly specified in Cochrane reviews by CRG in 2007, 2011, and 2013 may assist COS development.

**Electronic supplementary material:**

The online version of this article (doi:10.1186/s13643-015-0060-0) contains supplementary material, which is available to authorized users.

## Background

The Core Outcome Measures for Effectiveness Trials (COMET) Initiative, launched in 2010, seeks to advance the development of core outcome sets (COS) to address problems of outcome heterogeneity and outcome reporting bias [1, 2]. COS are minimum sets of outcomes that should be measured in any trial for a specific condition of interest, thus promoting consistency in the availability of information of the effects of interventions on key outcomes [3]. The COMET Initiative aims to link the development and use of COS with the specification of outcomes for Cochrane reviews, including the outcomes listed in their summary of findings (SoF) tables. The Cochrane Collaboration is a not-for-profit organisation that produces systematic reviews (Cochrane reviews), which summarise information from individual studies to inform health decisions [4]. Organisationally, it is structured into Cochrane Review Groups (CRGs) that are supported by a central administration. CRGs are disease and health condition-focused groups that work with Cochrane review authors and editors to develop Cochrane reviews. Summary of findings tables were developed by Grading of Recommendations Assessment, Development and Evaluation (GRADE) and were introduced into Cochrane reviews in 2008. These tables include a summary of the evidence for important outcome(s), as selected by the review authors, and the quality of this evidence [5]. They improve readers’ understanding and speed of retrieval of the findings of Cochrane reviews, and thus, inclusion of the most relevant outcomes in these tables is important [6, 7]. As COS are key outcomes that should be measured, they may provide a useful way to select outcomes to include in SoF tables. In a survey of CRG co-ordinating editors, 73 % suggested that COS should be used in SoF tables [8].

As part of our work within the COMET Initiative, we performed an initial exploratory survey of outcomes in Cochrane reviews that were published in full for the first time in 2007 and 2011 [9]. This survey explored the variety of outcomes used in these Cochrane reviews and the use of COS in reviews on specific conditions. The survey also identified the proportion of outcomes specified in the methods section that was reported in the results section of the Cochrane reviews and assessed the uptake of SoF tables in 2011 Cochrane reviews. Outcomes were examined by CRGs as a meaningful way to explore COS and outcome reporting in Cochrane reviews. The results of this initial survey showed that 37 % (1996/5363) of outcomes specified in the methods section of 702 Cochrane reviews that had studies in them were not reported in the results section of these reviews. For 14 % (732/5363) of cases, we did not find any reason in the text of the review for not reporting the outcome(s) in the review. None of the Cochrane reviews explicitly referred to a COS when specifying outcomes, and 31 % of 361 newly published 2011 Cochrane reviews that had studies in them had a SoF table. To determine possible changes in the use of COS in Cochrane reviews and changes in outcome reporting over time and to explore consistency in outcomes specified in reviews of individual CRGs (defined as being specified repeatedly by at least half of the new reviews in the individual CRGs in 2007, 2011, and 2013), we conducted a follow-up survey using Cochrane reviews published in full for the first time in 2013. As for the initial survey, this follow-up survey was concerned with examining what outcomes were examined in Cochrane reviews, rather than how these outcomes were measured.

### Aim

The aim of this study was to survey the outcomes used in newly published 2013 Cochrane reviews as a follow-up to the survey of outcomes in newly published 2007 and 2011 Cochrane reviews [9].

### Objectives

1. To identify and highlight the use of COS in reviews from CRGs
2. To identify the number and variety (i.e. different types) of outcomes specified and reported in these Cochrane reviews by CRG
3. To identify the inclusion of SoF tables in these Cochrane reviews by CRG, including where SoF tables are being used, to examine the number of outcomes included in them and the quality of evidence (GRADE) for these outcomes
4. To compare the findings for objectives 1–3 with the findings of the initial survey of 2007 and 2011 newly published reviews

## Methods

We conducted a descriptive survey of Cochrane reviews published in 2013 between March and August 2014. This follow-up survey was planned at the time of the initial survey to identify changes over time. The sample of Cochrane reviews in this repeat survey were thus published 5 and 2 years following the years of publication of the Cochrane reviews included in the initial survey, 3 years after the launch of the COMET Initiative, and 5 years after the introduction of SoF tables in Cochrane reviews.

We identified Cochrane reviews published in full for the first time in 2013 from the Cochrane repository Archie and retrieved full texts from the Cochrane Database of Systematic Reviews. Data that were extracted from each Cochrane review are presented in Table 1, and we constructed a separate data extraction table for each CRG. FW conducted the data extraction using the same template and guidelines that had been used by the researcher who had conducted data extraction for the initial survey (VS) [9] to ensure consistency in how outcomes were counted. Some of these guidelines, which are described further below, given the sheer variety in how outcomes were specified and the difficulty, in some instances, in identifying a clear alignment of the outcome to a specific category, were challenging to create. For this reason, in some cases, the ‘rules’ around extracting, counting, and categorising of outcome types were based on considerable discussion and consensus. For example, an outcome like ‘hospitalisation due to adverse effects’ could arguably be aligned to one of two major categories such as the domains of ‘hospital’ or ‘adverse events’. To handle these types of outcomes, a decision was reached, through discussion and consensus, that the main ‘stem’ of the outcome, that is the first appearing major domain within the outcome type, would indicate the alignment of the outcome, in the example’s case, ‘hospitalisation’. The following guidelines, to ensure consistency across surveys, were used to handle counting of outcomes in the Cochrane reviews. Specified outcomes in each review were identified by counting the number of separately listed outcomes in the methods section of the review. Where an outcome was divided further into sub-outcomes, it was still counted as 1 outcome. For example, if the outcome ‘adverse events’ was specified, but, the review authors also provided a list of examples of possible associated adverse events, we referred to ‘adverse events’ as the main outcome and to the listed examples of possible adverse events as sub-outcomes. Only the main outcome was counted in this case. Similarly, as the survey focused on ‘what’ outcomes were being measured and not ‘how’ they were measured, we did not count the different methods of measuring the same outcome as separate outcomes, rather the main outcome was counted once only. For example, if the outcome ‘pain’ was listed in the methods section of a review, and the review authors additionally specified pain as measured by visual analogue scale or numerical pain rating scale, we counted the outcome ‘pain’ as one outcome, based on outcome type, and not as two based on different measurement methods. The numbers of outcomes reported in the results section of reviews were similarly counted.

**Table 1:** Data that was extracted from included Cochrane reviews

• The number of included studies in the review
• The outcomes specified in the methods section of the review
• The outcomes reported in the findings section of the review (either narratively, numerically (e.g. using a risk ratio, odd ratio, risk difference) or in the meta-analysis)
• Possible reasons for not reporting an outcome in the review, for example when an outcome specified in the review had not been examined or reported in the studies included in that review
• Additional outcomes that were reported but that had not been specified in the methods section
• Inclusion of a SoF table in the review
• The number of outcomes in the SoF table (if applicable)
• The quality of evidence (GRADE) for the outcomes in the SoF table (if applicable)^a^

^a^If outcomes presented at more than one follow-up time point or for more than one comparison had different quality grades (which was the case in 24 SoF tables), the lowest quality grade was counted for that outcome

In the initial survey, 15 outcome categories had emerged from a scoping overview of the extracted data across the CRGs. These categories “Rather than attempting to define outcome domain systems, we used to extract and manage the data for analyses purposes” ([9], p. 239). In this sense, these categories are not definitive domains, nor do we mean them to be; rather, they are used in this survey to describe the types of categories of outcomes that are being used in Cochrane reviews and the variety of the types of individual outcomes that are reflective of these categories. These categories were: adverse events or effects (AE), mortality/survival, infection, pain, psychosocial, quality of life, activities of daily living (ADL), medication, economic, hospital, operative, compliance (with treatment), withdrawal (from treatment/study), satisfaction (patient/clinician/other healthcare provider), and other physiological or clinical (other than adverse events/effects, mortality/survival, pain, and operative outcomes). In this repeat survey, outcomes specified in the included reviews across all CRGs were categorised using these same categories so that comparisons to the initial survey could be made.

We used descriptive statistics to analyse the data. Spearman’s correlation was used to assess any link between the number of outcomes specified in a Cochrane review and the proportion of outcomes that were reported in the results section of the review and in the SoF table.

## Results

We identified 453 Cochrane reviews published in 2013 from 51 CRGs. After excluding 8 Cochrane reviews of diagnostic studies, 3 Cochrane overviews of reviews, 1 Cochrane review of qualitative studies, and all (2) Cochrane reviews by the methodology CRG, we included 439 Cochrane reviews from 50 CRGs in this survey. None of the Cochrane reviews stated explicitly that they used a COS in specifying their review outcomes.

### Number of specified outcomes in reviews

The number of specified outcomes in each Cochrane review ranged from 1 (3 reviews) to 62 (1 review), with a median of 7 outcomes, and an interquartile range of 5–10 outcomes. Of the 3 Cochrane reviews that specified 1 outcome only, 1 review assessed a specific drug adverse effect [10], 1 review assessed interventions for smoking cessation [11], and 1 review examined the effectiveness of a drug for a specific type of poisoning with mortality as its only outcome [12]. The review that specified 62 outcomes assessed different types of dietary advice to women with gestational diabetes and specified short and long-term outcomes for both mother and child [13].

**Fig. 1:**
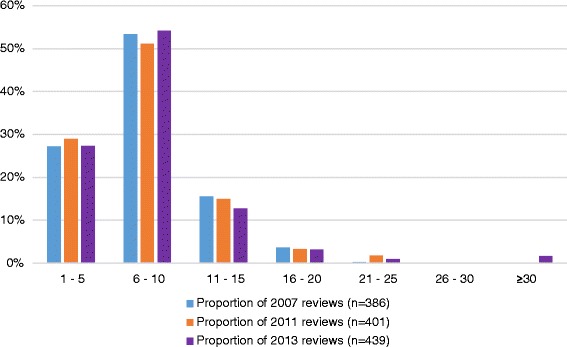
Number of specified outcomes (proportions) in 2007, 2011, and 2013 Cochrane reviews

Next, we compared the distribution of the number of specified outcomes in Cochrane reviews published in 2013 with the number of outcomes specified in Cochrane reviews published in 2007 and 2011 [9] (Fig. 1). Most Cochrane reviews published in 2007, 2011, and 2013 specified between 6 and 10 outcomes, whereas very few Cochrane review specified more than 20 outcomes.

### Reporting of specified outcomes

**Fig. 2:**
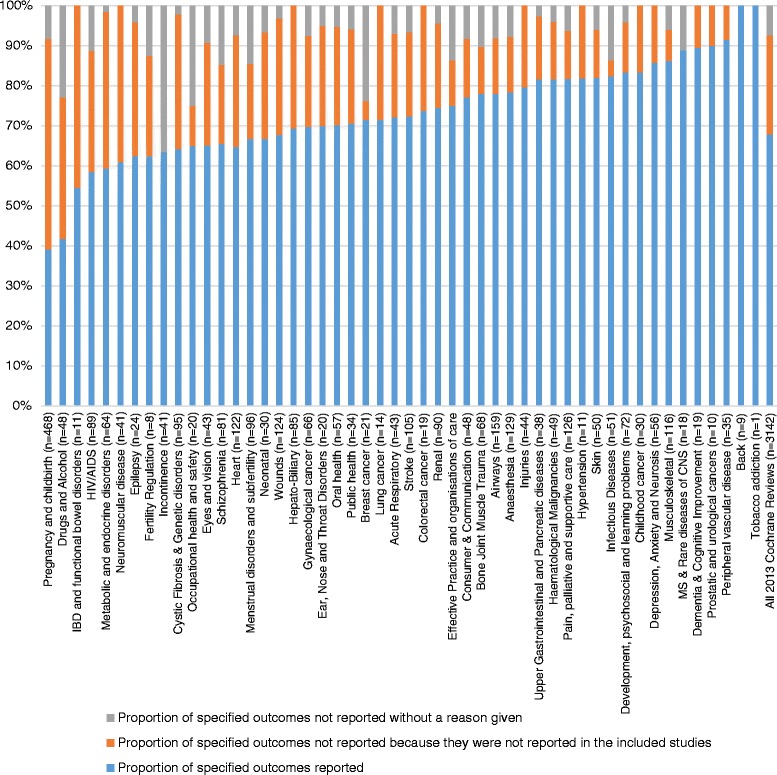
Specified outcomes reported and not reported by Cochrane Review Group: all outcomes

The 439 included Cochrane reviews specified a total of 3644 outcomes in their methods sections. After excluding Cochrane reviews that had no studies in them (65 reviews), 68 % (2134/3142) of the specified outcomes were reported in the results section of the reviews. Of the 1008 non-reported outcomes across all Cochrane reviews that had studies in them, 77 % (775/1008) were not reported in the reviews’ results sections because the outcomes had not been reported in the studies included in the reviews or the review authors had not been able to extract the relevant data for that outcome. However, for the remaining 23 % (233/1008) of outcomes not reported in the Cochrane reviews, we did not find a clear reason in the text of the review for this non-report (Fig. 2). In 12 Cochrane reviews, a total of 58 outcomes were noted in the review as being added post-hoc to the protocol. Of these 58 outcomes, 15 had been added to the list of specified outcomes in the methods section of these reviews by the authors. The remaining 43 were only present in the results section of the review.

There was a weak negative correlation between the number of outcomes specified and the proportion of the specified outcomes that were reported (Spearman’s correlation −0.3; *p* < 0.0001); Cochrane reviews that specified a larger number of outcomes in their methods section reported a smaller relative proportion of these outcomes in the results compared to Cochrane reviews that specified a smaller number of outcomes in their methods section. However, there was no correlation between the number of outcomes specified and the number of non-reported outcomes that did not have a clear reason for the non-report (Spearman’s correlation 0.079; *p* = 0.129).

When we examined primary outcomes only, we found that 84 % (698/829) of the specified primary outcomes were reported in the results section of the Cochrane reviews (range 22 % (1 CRG) to 100 % (16 CRGs) and median 89 % across CRGs). Eighty-nine percent of the 131 non-reported primary outcomes were not reported because the included studies did not either measure or report them. For the remaining 11 % of non-reported primary outcomes, we did not find any reasons in the text of the reviews for why these outcomes were not reported.

### Outcome variation/consistency within Cochrane review groups

There was considerable variation in the outcomes specified in the Cochrane reviews of the individual CRGs, particularly in the category ‘other physiological/clinical’.

Additional file 1 shows the outcomes that were specified in at least half the 2013 reviews for each CRG and makes comparison with the 2007 and 2011 Cochrane reviews [9]. The outcome ‘adverse events’ was specified in ≥50 % of reviews for 38 of the 50 CRGs, ‘quality of life’ was specified in ≥50 % of reviews for 17 of the 50 CRGs, and the outcome ‘mortality’ was specified in ≥50 % of reviews for 16 of the 50 CRGs. These were the 3 most commonly specified outcomes. Excluding CRGs with 2 or fewer new reviews in 2013 (because all specified outcomes are by default present in ≥50 % of reviews), the outcome ‘adverse events’ was specified in ≥50 % of reviews for 33 of 42 CRGs, the outcome ‘mortality’ was specified in ≥50 % of reviews for 15 of 42 CRGs, and ‘quality of life’ was specified in ≥50 % of reviews for 13 of 42 CRGs.

### Summary of findings tables

Fifty-seven percent (216/375) of the Cochrane reviews that had studies included in them had a SoF table. Three Cochrane reviews that did not have any included studies also provided an empty SoF table, but these were excluded from the analysis. Of the 216 Cochrane reviews with a SoF table, 12.5 % (27/216) included 1 outcome in the table, 16.2 % (35/216) included 2 outcomes, 14.4 % (31/216) included 3 outcomes, 12.5 % (27/216) included 4 outcomes, 17.1 % (37/216) included 5 outcomes, 11.6 % (25/216) included 6 outcomes, and 14.8 % (32/216) included 7 outcomes. In addition, 1 Cochrane review had 8 outcomes and a second had 9 outcomes in their SoF tables. The number of outcomes in the SoF table was positively correlated with the number of outcomes specified in the methods section of the review (Spearman’s correlation 0.468, *p* < 0.0001). Sixty-nine of the 216 Cochrane reviews with a SoF table included sub-outcomes for at least 1 outcome in the SoF table. Furthermore, in 8 reviews, the SoF table listed outcomes for more than 1 follow-up period, and 8 reviews included outcomes for more than 1 intervention comparison. One review also presented outcomes for 2 different populations within the SoF table, and another review listed the outcomes separately according to the design of the studies.

The quality of evidence (GRADE) for the results for the outcomes listed in the SoF tables varied with a ‘low quality’ grade being the most common grade (30 %; 266/877 outcomes in all SoF tables). Twenty-one percent (181/877) of effect size estimates in SoF tables were based on ‘very low quality’ evidence, 20 % (178/877) on ‘moderate quality’ evidence, and 8 % (73/877) had a ‘high quality’ grade. For the remaining outcomes listed in the SoF tables (179/877), the outcomes had not been either measured or reported in the included studies (118/877), insufficient data were present to estimate an effect size (22/877), or there was no reason provided for not reporting a quality grade (29/877). One Cochrane review used a different evidence quality grading system (strong/moderate/limited/conflicting/inconclusive/no evidence).

**Fig. 3:**
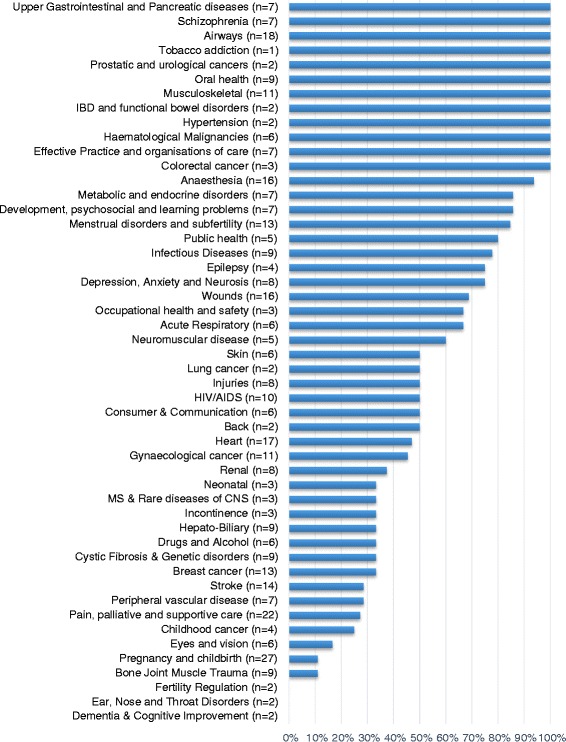
Proportion of reviews with a summary of findings table by Cochrane Review Group

The proportion of Cochrane reviews with SoF tables within individual CRGs ranged from 0 % (3 CRGs) to 100 % (12 CRGs) with a median of 50 % (Fig. 3).

## Discussion

In this follow-up survey of outcomes in Cochrane reviews, we found that the numbers of specified outcomes in the reviews were proportionately similar for 2007, 2011, and 2013 newly published Cochrane reviews, and the median number of outcomes specified in the methods section of the reviews was 7 for all years [9]. However, the maximum number of outcomes was only 26 in 2007 and 2011, whereas 7 Cochrane reviews from 2013 specified a greater number of outcomes. Although larger numbers of specified outcomes were not related to non-reporting in the absence of a clear reason being given for this non-report, the selection of outcomes for Cochrane reviews should receive careful consideration [9].

In this follow-up survey, 68 % of all specified outcomes were reported in the results section of the Cochrane reviews. This is an increase when compared to the 63 % of specified outcomes that were reported in 2007 and 2011 Cochrane reviews [9]. Moreover, 7 % of all specified outcomes were not reported, and we did not find any reason for this non-report in the text of these Cochrane reviews in 2013. This compares favourably to the earlier survey that found that 14 % of specified outcomes were not reported without reasons given for this non-report in the text of these Cochrane reviews [9]. Outcome reporting bias, that is, the selection of reported outcomes based on the outcome results, may significantly impact on the findings of studies and Cochrane reviews [1]. This observed reduction (50 %) over time in non-reporting of outcomes with no reason provided is welcome. Specified primary outcomes were also more frequently reported in 2013 than in the earlier survey, which is reassuring. However, in this survey, we compared the outcomes reported in the methods section of the Cochrane reviews with the outcomes reported in the results section. Ideally, the outcomes specified in the protocols would be compared with the outcomes reported in the results section of the completed Cochrane reviews; however, this was beyond the scope of the survey and is recognised, by the authors, as a limitation.

### Outcome variation/consistency within Cochrane review groups

Considering the findings for the specified outcomes within each CRG (Additional file 1), 48 of the 50 CRGs had at least 1 outcome that was specified in more than half of its reviews. This could be seen as an increase in consistency in the specified outcomes within CRGs (i.e. outcomes that are specified repeatedly in at least half of the reviews of an individual CRG) compared to 2007 and 2011, when only 41 of 50 CRGs had at least 1 outcome specified in more than half of its reviews. However, caution should be taken when considering this finding as 7 CRGs published 2 or fewer new reviews in 2013, meaning that all specified outcomes for these CRGs must have been specified in at least half of their reviews.

The slight increase in CRGs that specified the outcome ‘adverse event/effects’ in at least half of the reviews from 71 % of CRGs in 2007 and 2011 to 76 % CRGs in 2013 is welcome. However, this is not yet in line with the Cochrane Handbook for Systematic Reviews of Interventions’ recommendation that all Cochrane reviews should try to include adverse effects of interventions [5].

Comparing the outcomes for each CRG that were specified in at least half of the CRGs’ reviews in 2013 to those published in 2007 and 2011 [9] in greater detail (Additional file 1), consistency (defined as specified repeatedly in the methods section of at least half of the reviews in the CRG) across time varied from 1 CRG having no single outcome present in at least 50 % of reviews in 2007, 2011 and 2013, to 2 CRGs that had identical outcomes present in at least 50 % of reviews for the reviews published in 2007, 2011, and 2013. The outcomes that appear to have been consistently specified in Cochrane reviews over the last six years could possibly be considered for inclusion in a preliminary COS. However, the variation in the outcomes used in the reviews of a CRG will depend on the scope of the individual CRG. Logically, CRGs with a broader scope of reviews, covering multiple areas of health care, are likely to have a wider variety of outcomes.

### Summary of findings tables

The guidance in the Cochrane Handbook for Systematic Reviews of Interventions strongly recommends that Cochrane reviews should present the most important outcomes in a SoF table [5]. There has been an increased uptake of SoF tables in Cochrane reviews over time, with 57 % of the 2013 Cochrane reviews including a SoF table, compared to 31 % in 2011 [9]. However, the uptake of SoF tables varies greatly across CRGs. This rise may be due to more CRG editors requesting it, as well as increased training amongst editorial staff and authors in producing SoF tables. For example, three Cochrane reviews that had no studies in them still included a SoF table. The corresponding CRGs appear to request a SoF table, as standard, for every review as all of their 2013 published reviews had a SoF table. With 73 % of surveyed CRG editors suggesting that COS should be used in a SoF table [8], the increasing trend of including a SoF table in Cochrane reviews could be an opportunity to promote the use of COS in Cochrane reviews. An examination of the Cochrane reviews for our survey, however, found that as yet, none of the included Cochrane reviews stated specifically that they were using a COS to guide the selection of their outcomes or their decisions on which outcomes to present in the SoF table.

In line with the recommendation of identifying a maximum of up to 7 outcomes for including in a SoF table [5], all but 2 reviews had 7 or fewer outcomes in their SoF table. Four reviews did not have a grade of the quality of the evidence for the outcomes in their SoF table, despite this being part of the standard content [5].

## Conclusions

The proportion of specified outcomes that were reported in Cochrane reviews that had studies in them increased over time, from 61 % in 2007 to 65 % in 2011 and 68 % in 2013. Importantly, 2013 Cochrane reviews that did not report specified outcomes were twice as likely to provide an explanation for this non-report (i.e. 14 % in the 2007 and 2011 Cochrane reviews combined, compared to 7 % in the 2013 Cochrane reviews). Ideally, to reduce the potential for selection and reporting biases, a legitimate reason should be given for why all non-reported specified outcomes in Cochrane reviews are not being reported.

None of the Cochrane reviews stated explicitly that they used a COS in deciding which outcomes to specify in the methods sections of their reviews or to include in their SoF tables, although they may have considered a COS without clearly saying that specified outcomes came from it. Data on outcomes that are repeatedly being specified in the Cochrane reviews of a CRG at the 3 time points of the initial survey and this repeat survey (2007, 2011, 2013) may be useful for COS developers when considering what outcomes to include in a preliminary set of outcomes for a COS. It can provide another source of information in addition to the involvement of key stakeholders in COS development [2].

There has been an increased inclusion of SoF tables in Cochrane reviews, but these were still absent from nearly half of 2013 Cochrane reviews. As the use of SoF tables continues to be promoted, development of COS could go hand in hand with an increasing use of SoF tables in Cochrane reviews.

## Additional file

Additional file 1:
**Consistency of outcomes specified in 2013 Cochrane Reviews compared to 2007 and 2011 Cochrane Reviews.** Outcomes that were specified in at least half of the reviews first published in 2013 are presented by CRG.

## Abbreviations

COMET: Core outcome measures for effectiveness trials
COS: Core outcomes set
CRG: Cochrane Review Group
SoF table: Summary of findings table
