# Supplementary material for: Towards core outcome set (COS) development: a follow-up descriptive survey of outcomes in Cochrane reviews
Source: Syst Rev. 2015 May 19;4:73. doi: 10.1186/s13643-015-0060-0 (PMC4489040; doi:10.1186/s13643-015-0060-0)
Supplement: Additional file 1: — Consistency of outcomes specified in 2013 Cochrane Reviews compared to 2007 and 2011 Cochrane Reviews. Outcomes that were specified in at least half of the reviews first published in 2013 are presented by CRG. [file 13643_2015_60_MOESM1_ESM.pdf]

Additional file 1: Consistency of outcomes specified in 2013 Cochrane Reviews compared to 2007 and 2011 Cochrane Reviews

**Review Group (n=number of reviews in CRG after excluding diagnostic studies, reviews of reviews)**	**Outcomes that were specified ≥50% of Reviews**	**Number of 2013 reviews specifying the outcome (%)**
Acute Respiratory Group (n=6)	Adverse events/effects * ~<br>Mortality (all-cause)<br>Costs	4 (67%)<br>4 (67%)<br>3 (50%)
Airways (n=19)	Adverse events/effects * ~<br>Symptom severity/control * ~<br>Lung function * ~<br>Exacerbations * ~<br>Quality of Life (generic and/or specific) ~	11 (58%)<br>11 (58%)<br>12 (63%)<br>12 (63%)<br>14 (74%)
Anaesthesia (n=17)	Mortality *	13 (76%)
Back (n=2)	*Only 2 reviews published in 2013, hence all outcomes are present in ≥50% of reviews.*	
Bone, Joint & Muscle trauma (n=10)	Adverse events/effects ~<br>Pain * ~<br>Function/disability *<br>Return to work/former activities ~	6 (50%)<br>7 (58%)<br>9 (75%)<br>8 (67%)
Breast Cancer (n=4)	Adverse event/effects *<br>Incidence of seroma<br>Volume of seroma aspirations<br>Quality of Life (generic) *	2 (50%)<br>2 (50%)<br>2 (50%)<br>3 (75%)
Childhood Cancer (n=5)	Adverse events/effects	3 (60%)
Colorectal Cancer (n=3)	Complications (intervention related)<br>Mortality (all cause)	2 (67%)<br>2 (67%)
Consumer & Communication (n=6)	Adverse events/effects<br>Knowledge/understanding ~<br>Organisational outcomes	4 (67%)<br>5 (83%)<br>6 (100%)
Cystic Fibrosis & Genetic Disorders (n=12)	Adverse events/effects * ~<br>Lung function<br>Nutritional status/anthropometry	11 (92%)<br>8 (67%)<br>6 (50%)
Dementia & Cognitive Improvement (n=3)	Adverse events/effects * ~<br>Mortality (all cause)<br>Global function * ~<br>Cognitive function<br>Quality of Life (generic) * ~	3 (100%)<br>3 (100%)<br>2 (67%)<br>2 (67%)<br>2 (67%)
Depression, Anxiety & Neurosis (n=8)	Adverse events/effects ~<br>Treatment response/efficacy<br>Quality of Life (generic) * ~<br>Treatment acceptability (drop-out) *	5 (63%)<br>4 (50%)<br>5 (63%)<br>4 (50%)
Development. Psychosocial & learning problems (n=8)	Adverse events/effects ~	5 (63%)
Drugs & alcohol (n=7)	Adverse events/effects<br>Mortality (all cause)<br>Drug use * ~	5 (72%)<br>4 (57%)<br>6 (86%)
Ear, Nose & Throat Disorders (n=2)	*Only 2 reviews published in 2013, hence all outcomes are present in ≥50% of reviews.*	

Effective Practice and organisation of care (n=7)	Adverse events/effects<br>Costs	4 (57%)<br>4 (57%)
Epilepsy (n=6)	Adverse events/effects ~<br>Seizure reduction (≥50%) * ~<br>Seizure free *<br>Quality of Life (generic) *<br>Withdrawals ~	6 (100%)<br>3 (50%)<br>5 (84%)<br>4 (67%)<br>4 (67%)
Eyes & Vision (n=10)	Adverse events/effects * ~<br>Visual acuity * ~<br>Quality of Life (generic) * ~	6 (60%)<br>8 (80%)<br>6 (60%)
Fertility Regulation (n=2)	*Only 2 reviews published in 2013, hence all outcomes are present in ≥50% of reviews.*	
Gynaecological Cancer (n=17)	Adverse events/effects<br>Overall Survival ~<br>Progression/disease-free survival ~	12 (67%)<br>12 (67%)<br>10 (56%)
Haematological Malignancies (n=6)	Adverse events/effects ~<br>Mortality (all cause) ~<br>Overall Survival ~<br>Progression/disease-free survival<br>Mortality (intervention-related)<br>Response rate<br>Quality of Life (generic)	6 (100%)<br>4 (67%)<br>3 (50%)<br>3 (50%)<br>3 (50%)<br>4 (67%)<br>3 (50%)
Heart (n=17)	Adverse events/effects ~<br>Mortality (all cause) * ~<br>Quality of Life (generic) ~<br>Costs	12 (71%)<br>10 (59%)<br>10 (59%)<br>11 (65%)
Hepato-Biliary (n=11)	Adverse events/effects * ~<br>Mortality (all cause) * ~<br>Quality of Life (generic) * ~	10 (91%)<br>10 (91%)<br>10 (91%)
HIV/AIDS (n=10)	Adverse events/effects ~<br>Mortality (all cause)	9 (90%)<br>7 (70%)
Hypertension (n=2)	*Only 2 reviews published in 2013, hence all outcomes are present in ≥50% of reviews.*	
Incontinence (n=4)	Adverse events/effect * ~<br>Urinary incontinence (subjective) * ~<br>Urinary incontinence (clinician observation)<br>Incontinence episodes<br>Pad changes<br>Pad test<br>Urodynamic measures * ~<br>Pelvic floor muscle function/strength<br>Incontinence improvement (subjective)<br>Quality of Life (generic) * ~<br>Quality of Life (specific) * ~<br>Quality of Life (generic or specific)	4 (100%)<br>3 (75%)<br>2 (50%)<br>3 (75%)<br>3 (75%)<br>4 (100%)<br>2 (50%)<br>2 (50%)<br>2 (50%)<br>2 (50%)<br>2 (50%)<br>3 (75%)
Infectious Diseases (n=9)	Adverse events/effects * ~<br>Mortality (all cause)	5 (50%)<br>6 (50%)
IBD & Function Bowel Disorders (n=2)	*Only 2 reviews published in 2013, hence all outcomes are present in ≥50% of reviews.*	
Injuries (n=11)	Mortality (all cause) ~	8 (73%)
Lung Cancer (n=2)	*Only 2 reviews published in 2013, hence all outcomes are present in ≥50% of reviews.*	
Menstrual Disorders & subfertility (n=16)	Adverse events/effects * ~<br>Live births ~	9 (53%)<br>9 (53%)

Metabolic & Endocrine Disorders (n=7)	Adverse events/effects * ~<br>Mortality (all cause) * ~<br>Quality of Life * ~<br>Costs * ~	7 (100%)<br>6 (86%)<br>7 (100%)<br>4 (58%)
MS & Rare Disease of the CNS (n=3)	Adverse events/effects * ~<br>Clinical relapse<br>Disability progression *	3 (100%)<br>2 (67%)<br>2 (67%)
Musculoskeletal (n=12)	Adverse events/effects * ~<br>Pain *<br>Fatigue	7 (54%)<br>10 (77%)<br>7 (54%)
Neonatal (n=6)	Adverse events/effects<br>Mortality (all cause) *<br>Neurodevelopment * ~	4 (67%)<br>5 (71%)<br>4 (57%)
Neuromuscular Disease (n=7)	Adverse events/effects * ~<br>Pain reduction ≥ 30%	7 (100%)<br>4 (57%)
Occupational Safety & Health (n=4)	Recurrence/relapse<br>Quality of Life (generic)<br>Functional status	2 (50%)<br>2 (50%)<br>2 (50%)
Oral Health (n=13)	Adverse events/effects * ~	7 (50%)
Pain, Palliative & Supportive Care (n=24)	Adverse events/effects * ~	17 (71%)
Peripheral Vascular Disease (n=11)	-	-
Pregnancy & Childbirth (n=35)	Perinatal/neonatal mortality *	28 (80%)
Prostatic & Urologic Cancers (n=2)	*Only 2 reviews published in 2013, hence all outcomes are present in ≥50% of reviews.*	
Public Health (n=5)	-	-
Renal (n=8)	Mortality (all cause) * ~	6 (75%)
Schizophrenia (n=10)	Adverse events * ~<br>Mental state * ~<br>Social Functioning<br>Quality of Life (generic) * ~<br>General Functioning<br>Service utilisation * ~<br>Economic outcomes * ~<br>Withdrawals* ~<br>Satisfaction with care * ~	7 (70%)<br>9 (90%)<br>5 (50%)<br>5 (50%)<br>5 (50%)<br>10 (100%)<br>7 (70%)<br>7 (70%)<br>6 (60%)
Skin (n=7)	Adverse effects * ~<br>Quality of Life *	5 (72%)<br>5 (72%)
Stroke (n=15)	Adverse events/effects * ~	11 (73%)
Tobacco Addiction (n=2)	*Only 2 reviews published in 2013, hence all outcomes are present in ≥50% of reviews.*	
Upper GI & Pancreatic Diseases (n=8)	Mortality (all cause) *	5 (56%)
Wounds (n=17)	Adverse events/effects ~<br>Quality of Life (generic) * ~<br>Length of hospital stay *	15 (88%)<br>12 (71%)<br>11 (65%)

* Categories of outcomes that were also specified in ≥50% of reviews published in 2007
~ Categories of outcomes that were also specified in ≥50% of reviews published in 2011
Cochrane Review Group in *italics* were not present in the initial survey (2007 and 2011), hence no comparison could be made.
